# The House of Steinway

**Published:** 1875-01

**Authors:** 


### THE HOUSE OF STEINWAY.

We desire to give our readers some distinct idea of the materials employed and the methods adopted in the construction of a Piano Forte at the great factory of Messrs. Steinway & Sons, in this city. It has well been said that to visit this establishment is a lesson in the noble art of taking pains. The construction of an American piano is a continual act of defensive warfare against the future inroads of our climate, a climate which is polar for a few days in January, tropical for a week or two in July, Nova Scotian now and then in November, and at all times most trying to the finer woods, leathers and fabrics. To make a piano is not very difficult; but to make one that will stand in America, that is very difficult. In the rear of Messrs. Steinways' factory there is a yard for seasoning timber, which usually contains an amount of material equal to two hundred and fifty thousand ordinary boards, an inch thick and twelve feet long, and there it remains from four months to five years, according to its nature. Most of the timber used in a piano requires not less than two years' seasoning. From the yard it is transferred to the steam-drying house, where it remains subjected to a high temperature for three months. The wood has then lost nearly all the warp that ever was in it, and the temperature may change fifty degrees in twelve hours—as it sometimes does in our Northern climate—without seriously affecting a fibre. Besides this, the timber is sawed in such a manner as to neutralize, in some degree, its tendency to warp, or rather so as to make it warp the right way. The reader would be surprised to hear these great makers converse on this subject of the warping of timber. They have studied the laws which govern warping; they know why wood warps, how each variety warps, how long a time each continues to warp, and how to fit one warp against another, so as to neutralize both. If two or more pices of wood are to be glued together, it is never done at random; but they are so adjusted that one will tend to warp one way, and another another. Even the thin veneers upon the case act as a restraining force upon the less ornamental wood which they cover, and in some parts of the instrument the veneer is double for the purpose of keeping both in order. An astonishing amount of thought and experiment has been expended upon this matter of warping—so much, that now not a piece of wood is employed in a Steinway piano, the grain of which does not run in the precise direction which experience has shown to be the best.

The forests of the whole earth have been and are to-day being searched for woods adapted to the different parts of the instrument. A catalogue of the various woods, metals, skins, and fabrics used in the construction of the modern piano, will forcibly illustrate the delicacy of the modern instrument and the infinite care taken in its manufacture. Of woods, we have the oak from Riga, which is used in various parts of the framing; the deal from Norway, employed as wood-bracing; the fir from Switzerland, which forms the sounding-board; the American pine, from which parts of framing, key-board, and bottom are made; the mahogany from Honduras, constituting the solid wood of top and various parts of the framing, and the action; the beech from England, for the wrest-plank, bridge or sound-board, and the centre of legs; the beef-wood from Brazil, used as tongues in the beam, forming the divisions between the hammers; the birch from Canada, from which the portion known as the "belly-rail" is made; the cedar from South America, for the round shanks of hammers; the lime-tree from England, for the keys; the pear-tree, used in the heads of dampers; the sycamore, for the hoppers or levers, and the veneers on the wrest-plank; the ebony from Ceylon, for the black-keys; whlie for inlaying and various decorative purposes, there are Spanish mahogany from Cuba; rosewood from Rio Janeiro; Satinwood from the East Indies; white-holly from England; zebra-wood from Brazil, and other fancy woods from far distant lands.

Of other substances, we have baize, for the upper surface of key-frame, as cushions for hammers to fall on, and as dampers for the dead portions of the strings; cloth of several kinds, for various parts of the action and in other places to prevent jarring; felt as the external covering of the hammers; buffalo leather for the under covering of the bass-hammers; saddle leather for the under covering of the tenor and treble hammers; basil leather, calf-skin, doe-skin, seal-skin, sheep-skin, morocco, all used in various parts of the action; sole-leather, as rings for the pedal-wires; iron, steel, brass and gun-metal, employed in the metallic frame-work, as well as in various screws, springs, centres, pins and connections throughout the entire instrument; steel-wire for strings; steel spun wire for lapped strings, and covered copper wire for lowest notes. Added to all these are the ivory for the white keys; the glue for the wood-work; the black-lead as a lubricant, and the beeswax, emery paper, glass paper, shellac, oil, spirits of wine and many other substances employed in cleaning and finishing the structure.

Such are the materials used. The processes to which they are subjected are far more numerous. So numerous are they and so complicated, that the Steinways, who employ six hundred men, and labor-saving machinery which does the work of over six hundred more, aided by three steam-engines of a hundred and twenty-five, fifty, and twenty-five horse-power, can only produce from fifty-five to sixty-five pianos each week.

Doubtless our readers have often seen a piano with the top removed, and have observed the fifty or sixty strings which supply the musical notes; but possibly they may not have given a thought to the enormous strain which those strings are keeping up day and night from one years' end to another. We alluded to this in a former chapter, but the subject may well be adverted to again. The shortest and thinnest of these strings pulls two hundred and sixty pounds; the aggregate lifting power exerted by the entire series of keys, when at concert pitch, is twenty tons. This strain is not chiefly upon the iron frame; it is expended upon an immense mass of timber so arranged that the pull of the wires shall be in a line with the grain of the wood. The iron frame itself is subjected to a long course of treatment. The rough casting is brought from the foundry, placed under the drilling machine, which bores many scores of holes, of various sizes with marvellous rapidity. Then it is smoothed and finished with the file; next it is japanned; after which it is baked in an oven for forty-eight hours. It is then ready for the bronzer and gilder, who cover the greater part of the surface with a light yellow bronzing and brighten it here and there with gilding. All this is necessary in order to make the plate retain its brilliancy of color. Of other processes, as we have witnessed them at the great works of the Steinways, we will speak next month.

